# Re-examining the strangest early vertebrate

**DOI:** 10.1093/nsr/nwaf021

**Published:** 2025-01-25

**Authors:** Per Erik Ahlberg

**Affiliations:** Department of Organismal Biology, Uppsala University, Sweden

**Figure 1.**
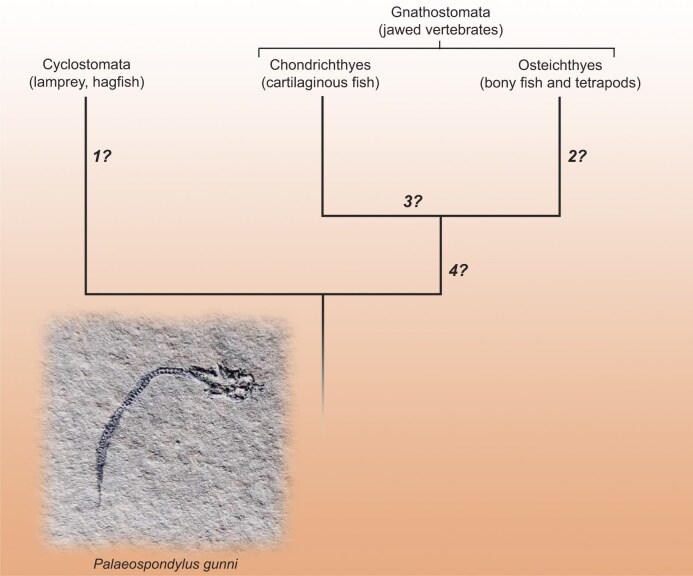
Consensus molecular phylogeny of living vertebrates, recovered by all recent analyses. Numbers with question marks represent possible placements of *Palaeospondylus* as: (1) a hagfish relative [2], (2) a stem tetrapod [3], (3) a basal chondrichthyan [1] or (4) a stem gnathostome [this paper]. Photo of the type species *Palaeospondylus gunni*, a specimen from Achanarras in Scotland. By Skye McDavid, reproduced with permission.

The early fossil record of vertebrates, from the Ordovician to Devonian periods (roughly 500 to 350 million years ago), is an Aladdin's Cave of strange treasures. The molecular phylogeny of the major living vertebrate groups (Fig. 1), the deep nodes of which lie within this timeframe, is straightforward: Gnathostomata (jawed vertebrates) form a clade with two major branches, Osteichthyes (bony fishes and tetrapods) and Chondrichthyes (cartilaginous fishes), and their joint sister group is the Cyclostomata (lampreys and hagfish). However, while some early fossil vertebrates can be readily placed on one or another of these branches, others are so bizarre that they defy classification even when their anatomy is well-preserved and informative. In this issue, Burrow and colleagues use new material from the Devonian of Australia to investigate one of the most mystifying of these creatures: *Palaeospondylus*, originally described in the 19th century from Scottish fossils [1]. They make progress with interpreting its anatomy, and are able to disprove some previous hypotheses about its relationships, but a robust positive identification remains elusive.

Until the present discovery, *Palaeospondylus* was known almost exclusively from the Scottish Middle Devonian (∼390 million years old) locality of Achanarras, which represents the deep part of a large tropical lake. Hundreds of the fossils have been found at this locality, each looking like two centimetres of black thread with a knot at one end; under the microscope, the ‘thread’ resolves into a vertebral column with a tail fin and the ‘knot’ into a skull with complex anatomy (Fig. 1). At even higher resolutions (achievable by physical thin sectioning or micro-CT scanning) the tissue is seen to consist of cartilage with preserved cell spaces. Unfortunately, the Achanarras fossils are strongly flattened and the strange anatomy is hard to understand. Recent interpretations have ranged all the way from a hagfish to a close relative of tetrapods; further progress has seemed impossible [2,3].

This is where the new Australian fossils come in. About 10 million years older than the Scottish material, these fossils are less complete, but fully three-dimensional. Freed from the enclosing rock with acid, a complete braincase and other isolated skull elements have been studied by SEM and micro-CT, and assembled into an interpretation of the skull that represents a huge improvement on our previous understanding. But what emerges is arguably the strangest vertebrate I have ever seen. It is clearly neither a hagfish nor a tetrapod ancestor.

Burrow *et al.* present a phylogenetic analysis that recovers *Palaeospondylus* as a basal chondrichthyan. However, they acknowledge that the character suite contains conflicting phylogenetic signals, and moreover may be affected by *Palaeospondylus* being either a larva or paedomorphic adult retaining larval characteristics. Some aspects of the anatomy differ from the ‘standard construction’ shared by all living jawed vertebrates and expressed in their embryo development: the notochord is not enclosed in the skull base, the supposed jaws are hard to understand, and the fissures separating the different constituent skull cartilages are not in the usual positions. All this suggests to me that *Palaeospondylus* could be something wholly outside our previous experience, perhaps a late survivor of a transitional grade at the origin of jawed vertebrates, below the last common ancestor of the living groups. It's an intriguing thought. However, given the unavailability of molecular and developmental data from this long-dead animal, we may never know; some fossils insist on keeping their secrets.
